# 抗PD-L1抗体的心脏毒性及甲状腺素的救治作用实验研究

**DOI:** 10.3779/j.issn.1009-3419.2021.102.23

**Published:** 2021-06-20

**Authors:** 振寅 陈, 敏 王, 三惠 高, 花 郭, 桂珍 王, 光飚 周

**Affiliations:** 1 550025 贵阳，贵州大学医学院 Guizhou University School of Medicine, Guiyang 550025, China; 2 100021 北京，国家癌症中心/中国医学科学院肿瘤医院分子肿瘤学国家重点实验室 State Key Laboratory of Molecular Oncology, National Cancer Center/National Clinical Research Center for Cancer/Cancer Hospital, Chinese Academy of Medical Sciences & Peking Union Medical College, Beijing 100021, China; 3 100101 北京，中国科学院动物研究所膜生物学国家重点实验室 State Key Laboratory of Membrane Biology, Institute of Zoology, Chinese Acadamy of Science, Beijing 100101, China

**Keywords:** 程序性细胞死亡受体配体1, 心脏毒性, 甲状腺素, Programmed cell death ligand 1, Cardiotoxicity, Thyroid

## Abstract

**背景与目的:**

免疫检查点抑制剂在肿瘤治疗中表现出了显著的疗效，但也可能会引起免疫相关的不良反应（immune-related adverse events, irAEs）。近来国际上报道了数百例免疫检查点抑制剂相关心肌炎，患者死亡率达50%。本研究目的是在动物水平重现临床情况，通过多次静脉注射抗程序性细胞死亡受体1（programmed cell death 1, PD-1）抗体、抗程序性细胞死亡受体配体1（programmed cell death ligand 1, PD-L1）抗体，观察药物对正常小鼠所产生的心脏毒性，并探讨L-甲状腺素的保护作用。

**方法:**

通过尾静脉向6周龄-8周龄的C57BL/6小鼠分别注射抗PD-1抗体（剂量为12.5 μg/g，每5天注射1次，共6次）、抗PD-L1抗体（10 μg/g，每周注射1次，共6次）、抗PD-L1抗体（用法用量同上）联合L-甲状腺素（注射抗体前30 min腹腔注射，剂量为0.25 μg/g）或同型对照免疫球蛋白IgG（10 μg/g，每周1次，共6次）。通过超声心动图检测小鼠心脏射血功能；通过无创鼠尾动脉测压仪、小动物生命体征监护仪检测小鼠血压、心电特征与体温；通过酶联免疫标记法检测小鼠血清中游离甲状腺素浓度。

**结果:**

小鼠心脏表达不同水平的PD-L1。12只接受同型对照免疫球蛋白、12只接受抗PD-1抗体的小鼠无一发生死亡。12只接受3次-6次抗PD-L1抗体注射的小鼠的心重胫骨比显著升高，心肌细胞出现变性、透明化及血管外炎症细胞浸润等改变。心脏超声及心电图检测结果显示，小鼠的心脏射血功能严重受损、心肌缺血。此外，小鼠注射抗PD-L1抗体后血压下降、体温异常降低，血清甲状腺素（tetraiodothyronine, T4）水平降至对照组的1/3。这些小鼠中有8只死亡，死亡率达66.7%。在注射抗PD-L1抗体前30 min经腹腔注射L-甲状腺素可明显降低小鼠死亡率。

**结论:**

抗PD-1抗体不会引起小鼠心脏毒性及小鼠死亡。抗PD-L1抗体可引起心脏毒性导致小鼠死亡，而L-甲状腺素有明显的保护作用。

## Abstract (en)

**Background and objective:**

Immune checkpoint inhibitors (ICIs) such as antibodies against programmed cell death 1 (PD-1) and programmed cell death ligand 1 (PD-L1), have shown remarkable efficacies in many subtypes of cancers. However, ICIs may also cause severe immune-related adverse events in the recipient patients. Recently, ICI-associated myocarditis have been reported in hundreds of patients worldwide, with a mortality rate of approximately 50% in these cases. This study aims to recapitulate the cardiotoxicity and explore the detoxicifying approaches to attenuate mortality caused by PD-1/PD-L1 inhibitors in healthy mice.

**Methods:**

Six to eight-week-old C57BL/6 mice were inoculated with anti-PD-1 antibody (12.5 μg/g every 5 days for 6 injections), anti-PD-L1 antibody (10 μg/g once a week for 6 weeks), anti-PD-L1 antibody (with the same dosage described above) in combination with levothyroxine (0.25 μg/g, intraperitoneally injected half an hour before anti-PD-L1 antibody injection), or isotype control immunoglobulin IgG (10 μg/g once a week for 6 weeks). The ejection function of the hearts was detected by echocardiography, body temperature and blood pressure were detected by Mouse Monitor^TM^ and non-invassive blood pressure minotor, and serum free thyroxine concentration was detected by The enzyme linked immunosorbent assay (ELISA).

**Results:**

PD-L1 was expressed at different levels by the cardiomyocytes of the mice. The isotype control immunoglobulin and anti-PD-1 antibody did not cause death of the mice. The 12 mice receiving 3-6 injections of anti-PD-L1 antibody showed a significant increase in the heart-to-tibial ratio and cardiomyoctye degeneration, hyalinization and extravascular inflammatory cell infiltration. In addition, the serum thyroxine was mardedly decreased to 1/3 of that in the control group mice, and the blood pressure and body temperature were abnormally decreased in mice upon treatment with PD-L1 blockade. Eight of the 12 (66.7%) mice died from multiple intravenous injection of anti-PD-L1 antibody.Intraperitoneal injection of levothyroxine 30 min before the injection of anti-PD-L1 antibody significantly attenuated the mortality rate of the anti-PD-L1 antibody-treated mice.

**Conclusion:**

The anti-PD-L1 antibody is cardiotoxic and lethal, and levothyroxine is able to rescue the mice from this immune checkpoint inhibitor-caused mortality.

免疫检查点是调控T细胞活性的负调节因子。通过免疫检查点抑制剂增强T细胞功能的肿瘤免疫治疗在罹患不同类型肿瘤的患者中都起到了显著的治疗效果。其中，以程序性细胞死亡受体1（programmed cell death 1, PD-1）和程序性细胞死亡受体配体1（programmed cell death ligand 1, PD-L1）为靶点的抗体在抗肿瘤治疗中的疗效较为显著^[1]^。PD-1/PD-L1抑制剂主要通过阻断肿瘤细胞与T细胞的结合，从而帮助T细胞有效识别肿瘤细胞并对其进行清除。研究^[2]^表明，应用PD-L1单抗Pembrolizumab在难治性原发性纵隔大B细胞淋巴瘤成人和儿童患者的有效率高达81%。

虽然免疫检查点抑制剂在肿瘤治疗中表现出了显著的疗效，但其在增强机体免疫功能的同时，也可能会引起免疫相关的不良反应（immune-related adverse events, irAEs）^[3]^。例如第一个被发现的免疫检查点细胞毒性T淋巴细胞相关蛋白4（cytotoxic T-lymphocyte-associated protein 4, CTLA-4），其抑制剂Ipilimumab于2011年被美国食品药品监督管理局（Food and Drug Administration, FDA）批准用于黑色素瘤的治疗^[4]^；但CTLA-4的阻断在激活次级淋巴器官中的T细胞时，会导致irAEs的发生。常见的不良反应如皮疹，发生率在43%-45%；消化系统不良反应如结肠炎、腹泻、肝炎；内分泌不良反应如下垂体炎、甲状腺功能减退等，也有着较高的发生率；而其他irAEs如肺炎、肾炎、神经系统和胰腺的不良反应发生率则相对较低^[5, 6]^。与之类似，PD-L1抑制剂在阻断PD-1/PD-L1的作用时，主要通过重新激活效应T细胞并促进其产生相应的细胞因子，发挥其抗肿瘤效应^[7, 8]^，因而对正常的免疫系统也有可能产生局部和区域性的负面影响。随着抗PD-1/抗PD-L1抗体在临床上的抗肿瘤应用越来越广泛，其导致的不良反应也有更多报道。Moslehi等^[9]^分析了世界卫生组织Vigibase数据库，报道了101例患者在应用免疫检查点抑制剂后发生严重心肌炎，且其中46例（46%）患者因之死亡。Al-Kindi等^[10]^利用FDA药品不良反应报告系统的数据，发现250例患者在应用免疫检查点抑制剂后发生心肌炎，且其中124例（50%）患者因之死亡。和其他抗肿瘤药物相比，免疫检查点抑制剂引起心肌炎的比例明显更高，且一些患者仅使用1剂-2剂的免疫检查点抑制剂即可发生致命性心肌炎^[9, 10]^。因此，在动物水平研究免疫检查点的心脏毒性及减轻毒性的方法，具有重要的实际意义。免疫检查点抑制剂已被广泛用于肺癌、黑色素瘤、结肠癌、肾癌、卵巢癌、胃癌、胰腺癌、乳腺癌等多种癌症患者的治疗^[11-13]^。抗PD-1、抗PD-L1抗体的治疗效果往往与PD-L1表达水平等有关，而其诱发的不良反应在上述所有类型的癌症患者中均有报道^[14]^。因此，在正常小鼠体内研究抗PD-L1抗体的毒副作用，可能更具代表性。本文以C57BL/6小鼠为动物模型，初步探索了多次尾静脉注射抗PD-1/抗PD-L1抗体后对小鼠心脏功能所能产生的不良反应及救治方法。

## 1 材料与方法

### 1.1 抗体与药品

抗PD1抗体购自Bio X Cell（BE0090, USA），在4 ℃冰箱避光保存，使用时用PBS稀释至2.5 μg/μL。抗PD-L1抗体购自Bio X Cell（BE0101, USA），在4 ℃冰箱避光保存，使用时用PBS稀释至2 μg/μL。同型对照IgG购自Bio X Cell（BE0273, USA），在4 ℃冰箱避光保存，使用时用PBS稀释至2 μg/μL。L-甲状腺素（Levothyroxine）购自Solarbio（T9621，北京），在4 ℃冰箱避光保存，使用时用1 mol/L NaOH溶液稀释至50 μg/mL。

### 1.2 动物实验

C57BL/6小鼠（雌性，5周龄-6周龄）和A/J小鼠（雌性，5周龄-6周龄）购自北京维通利华实验动物科技有限公司，在无特异病原体环境（specific pathogen free, SPF）饲养1周左右，称重，体重（20±0.5）g。分组予抗PD-1抗体或抗PD-L1抗体处理。实验方法遵循中国科学院动物所动物管理与使用委员会批准的指南进行操作。

#### 1.2.1 抗PD-1抗体

取12只C57BL/6小鼠，通过尾静脉注射抗PD-1抗体，剂量按小鼠体重计算为12.5 μg/g（平均为250 μg/只），每5天注射1次，共注射6次（表 1），观察小鼠存活情况。

**表 1:** 抗PD1、抗PD-L1抗体对小鼠生存的影响 Effect of anti-PD-1 and anti-PD-L1 antibodies on the survival of mice

Drugs	Dosage	Number of mice	Mice died from treatment	Mortality rate
Pre-experiments				
Anti-PD-1 antibody	12.5 μg/g, once every 5 days for 30 days	12	0	0.0%
Anti-PD-L1 antibody	5 μg/g, once a week for 6 weeks	7	5	71.4%
Anti-PD-L1 antibody	10 μg/g, once a week for 6 weeks	6	4	66.7%
Anti-PD-L1 antibody	15 μg/g, once a week for 6 weeks	5	4	80.0%
Formal experiments				
Isotype IgG-control	10 μg/g, once a week for 6 weeks	12	0	0.0%
Anti-PD-L1 antibody	10 μg/g, once a week for 6 weeks	12	8	66.7%
Anti-PD-L1 antibody+levothyroxine (T4)	Anti-PD-L1 antibody at 10 μg/g, once a week for 6 weeks; Levothyroxine, 0.25 μg/g, intraperitoneally injected 0.5 h before anti-PD-L1 antibody injection	10	0	0.0%
PD-1: programmed cell death 1.				

#### 1.2.2 抗PD-L1抗体

预实验：小鼠分为3组，分别接受抗PD-L1抗体尾静脉注射，剂量分别为5 μg/g、10 μg/g、15 μg/g，每周注射1次，共注射6次，观察小鼠存活情况（表 1）。根据小鼠存活情况及临床患者用量，确定10 μg/g每周注射1次共注射6次的剂量用于正式实验。正式实验：34只C57BL/6小鼠分为3组，第一组接受同型对照IgG尾静脉注射（*n*=12），剂量为10 μg/g每周注射1次共注射6次；第二组接受抗PD-L1抗体尾静脉注射（*n*=12），剂量为10 μg/g每周注射1次，共注射6次。以体表面积比折算，所使用抗PD-L1抗体剂量相当于成年人以1.11 mg/kg用量给药；第三组为抗PD-L1抗体与L-甲状腺素联合应用组（*n*=10），先腹腔注射L-甲状腺素0.25 μg/g，30 min后注射抗PD-L1抗体，后者的用法与用量同第二组。

在注射抗PD-L1抗体后，通过无创鼠尾动脉测压仪BP-98A（Softron, Japan）检测小鼠的心率和血压；通过Vevo 770 High-Resolution Imaging System仪器（Visual Sonics公司）进行小鼠心脏超声检测分析；通过Mouse Monitor^TM^实时小动物生命体征监护仪进行小鼠心电图（electrocardiogram, ECG）检测分析。小鼠死亡后收集心脏组织，于-80 ℃保存待用。

### 1.3 苏木精-伊红（hematoxylin-eosin, HE）染色

将福尔马林固定、石蜡包埋的小鼠心肌组织标本切成厚5 μm的切片，通过二甲苯脱蜡和不同浓度的乙醇对切片进行脱蜡处理。随后用苏木精染色5 min后，用盐酸酒精分化后，用伊红染液染色1 min-3 min；经过不同浓度梯度的酒精与二甲苯彻底脱水透明后，用中性树胶封盖，在普通光镜下观察组织形态。

### 1.4 酶联免疫标记

小鼠血液在室温自然凝固4 h，3, 000 rpm离心20 min，收集上层血清，用样品稀释液稀释5倍后，取50 μL加入包被小鼠游离甲状腺素（tetraiodothyronine, T4）抗体的微孔板（DUMA biotech，上海，中国），并在每孔中加入100 μL辣根过氧化物酶（horseradish peroxidase, HRP）标记的检测抗体，用封板膜封住反应孔，37 ℃孵育1 h。孵育完成后，弃去孔内液体，每孔加满洗涤液，静置1 min后弃去洗涤液，如此洗板5次后，每孔加入50 μL底物，在37 ℃下避光孵育15 min。之后每孔加入终止液50 μL，15 min内在450 nm波长处测定各孔OD值，与标准曲线比较得到小鼠血清内的T4浓度。

### 1.5 Western blot

将健康C57BL/6小鼠用颈椎脱臼的方式处死，取出小鼠心脏组织，对组织进行研磨后，用含蛋白酶抑制剂的RIPA裂解液（50 mmol/L TrisHCl pH 7.4、150 mmol/L NaCl、0.1%SDS、1%脱氧胆酸钠、1%TritonX-100、1 mmol/L EDTA、5 mmol/L NaF、1 mmol/L钒酸钠和Cocktail蛋白酶抑制剂）在冰上对研磨后的组织裂解30 min，12, 000 rpm离心5 min后弃去沉淀并通过BCA法对蛋白提取物进行定量。蛋白质（20 μg）经10%-15%的SDS-PAGE电泳后转移到硝化纤维素膜上。用5%脱脂牛奶/Tris缓冲液封闭1 h后，用TBST洗膜。所使用的anti-Mouse-PD-L1一抗购自Cell Signaling Technology公司（Beverly, MA, USA），anti-GAPDH一抗购自Sigma公司（St.Louis, MO, USA）。经一抗与二抗的孵育后用LSA 4000（GE, Fairfield, CO, USA）检测目标蛋白表达情况。

### 1.6 免疫组织荧光

将健康C57BL/6小鼠的心脏组织用组织冰冻切片OTC包埋剂（Sakura Finetek公司，Tokyo，Japan）进行包埋，之后用冷冻切片机（Leica Biosystems, Buffalo Grove, USA）对包埋样品进行切片，切片厚度为10 μm。所获得的切片经0.3%Triton X-100/PBS室温破膜处理20 min，之后用5%BSA封闭1 h。切片与anti-PD-L1一抗于4 ℃孵育过夜，用PBS清洗切片后加入Alexa Flour 488/647标记的二抗（Life technologies），室温孵育1 h。之后清洗切片，用含DAPI的封片剂封片后保存于4 ℃。图像由激光扫描共聚焦显微镜（N-STOR, Nikon, Japan）拍摄。

### 1.7 统计学分析

除非特殊说明，所获得的结果均用均数±标准差（Mean±SD）表示；采用GraphPad Prism 8.0软件做图；采用双尾Student's *t*检验方法对实验结果进行数据分析。*P* < 0.05表示差异有统计学意义。

## 2 结果

### 2.1 PD-L1在小鼠心脏组织中有较高的表达水平

PD-L1在黑色素瘤、肾细胞癌、肺癌、结肠癌、乳腺癌、卵巢癌、胃癌、头颈癌、恶性淋巴瘤、多发性骨髓瘤等多种肿瘤类型中有较高的表达，临床上使用抗PD-L1抗体治疗这些癌症患者也表现出了良好的疗效。PD-L1在正常组织中也有一定水平的表达。首先，我们通过Western blot检测了小鼠心脏组织中的PD-L1的表达水平，并通过光密度扫描且以GAPDH为参照对PD-L1表达水平进行相对定量。结果发现，PD-L1在小鼠心脏组织中有着较高的表达水平，且在不同小鼠个体之间有一定（约2.5倍）的差异（图 1A）；免疫组织荧光成像结果显示，PD-L1蛋白在心肌细胞中有着较为广泛的分布（图 1B）。以上这些结果表明，PD-L1在小鼠心脏组织中有着较高的表达水平。

**图 1:**
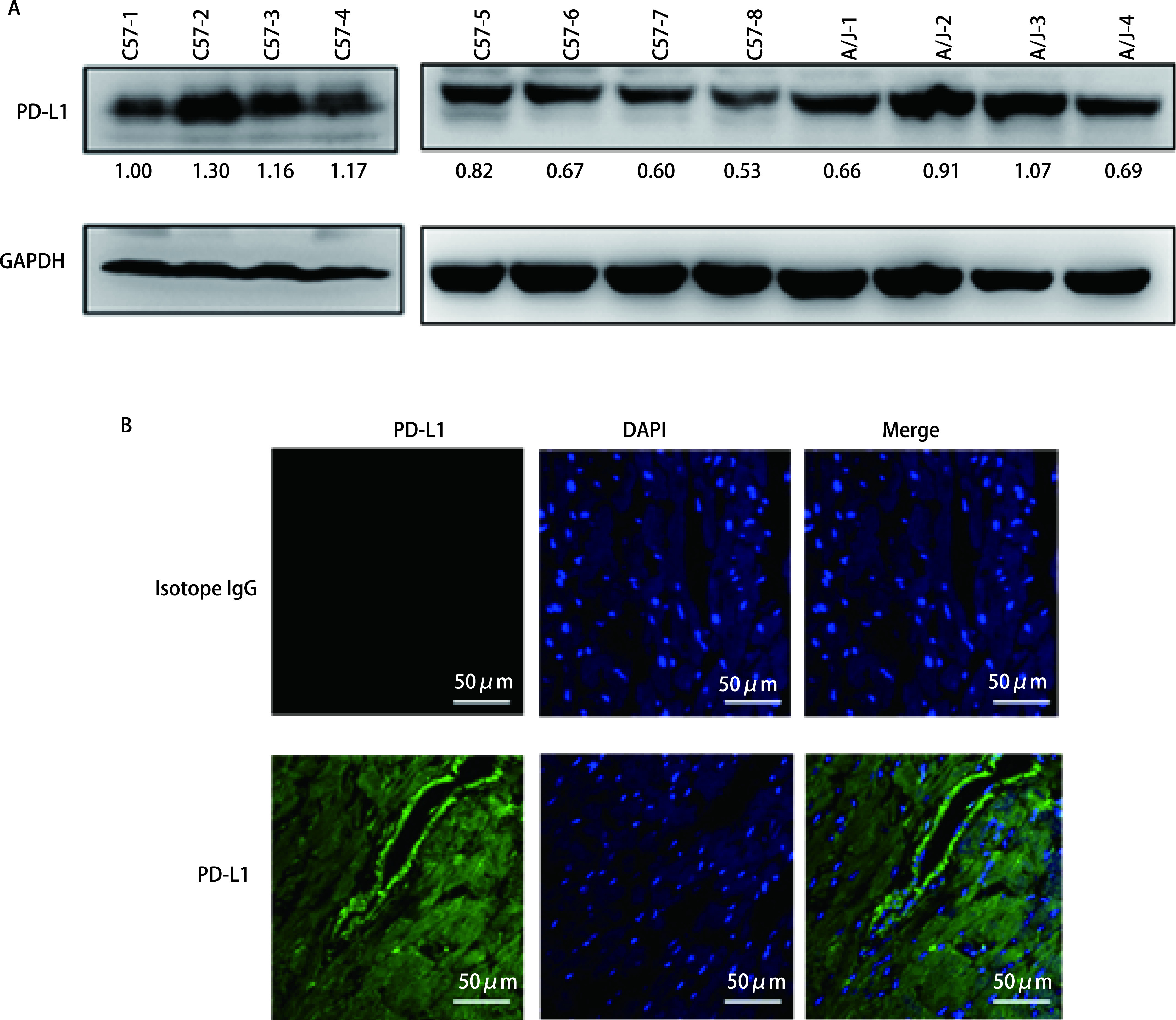
PD-L1在健康小鼠的心脏组织中高表达。A：Western blot检测小鼠心脏组织中的PD-L1表达水平。PD-L1条带下的数字为光密度扫描并以GAPDH为参照的PD-L1相对定量值；B：用免疫组织荧光检测小鼠心肌组织中PD-L1表达情况。 PD-L1 is highly expressed in heart tissues of healthy mice. A: PD-L1 expression levels in mouse heart tissues were detected by Western blot. Numbers under Western blot bands are the relative expression values to GAPDH determined by densitometry analysis; B: PD-L1 expression in mouse myocardial tissues was detected by immunohistochemical fluorescence. PD-L1: programmed cell death ligand 1.

### 2.2 抗PD-1抗体不引起小鼠死亡

我们通过尾静脉注射，用抗PD-1抗体处理小鼠，在250 μg/只、每5天注射一次连续注射6次的条件下，所有12只小鼠均未见明显异常，且在最后一剂抗体注射后2周未见小鼠死亡（表 1），说明抗PD-1抗体在小鼠是安全的。

### 2.3 多次静脉注射抗PD-L1抗体能引起小鼠的死亡

由于健康小鼠心脏组织中PD-L1表达水平较高，当小鼠注射PD-L1抑制剂后，其心脏组织有可能会受到一定程度的影响。因此，我们通过多次尾静脉注射抗小鼠PD-L1抗体至小鼠体内，剂量为10 μg/g（约为200 μg/只），每周注射1次，首先观察了PD-L1抑制剂对小鼠的状态和生存的影响。结果表明，当连续给予3次-4次抗小鼠PD-L1抗体后，小鼠状态表现为蜷缩不动，毛发蓬松脏乱无光泽，对外界刺激反应明显减弱、迟缓；当连续给予3次以上抗小鼠PD-L1抗体后，小鼠出现了死亡（图 2A），死亡率为8/12（66.7%）。注射相同剂量的同型对照IgG并不引起小鼠死亡（表 1）。分离死亡小鼠心脏，并对其称重后发现，注射抗PD-L1抗体组的小鼠心脏/体重比值和心脏/胫骨比值均显著高于IgG组小鼠（图 2B和图 2C）。而组织切片检查发现，抗PD-L1抗体处理组心肌细胞的形态并非常见的梭形，而表现为肥大的形态，并出现透明化，血管外可见淋巴细胞与中性粒细胞浸润（图 2D）。以上结果提示多次静脉注射抗PD-L1抗体能明显引起小鼠心脏组织毒性反应。

**图 2:**
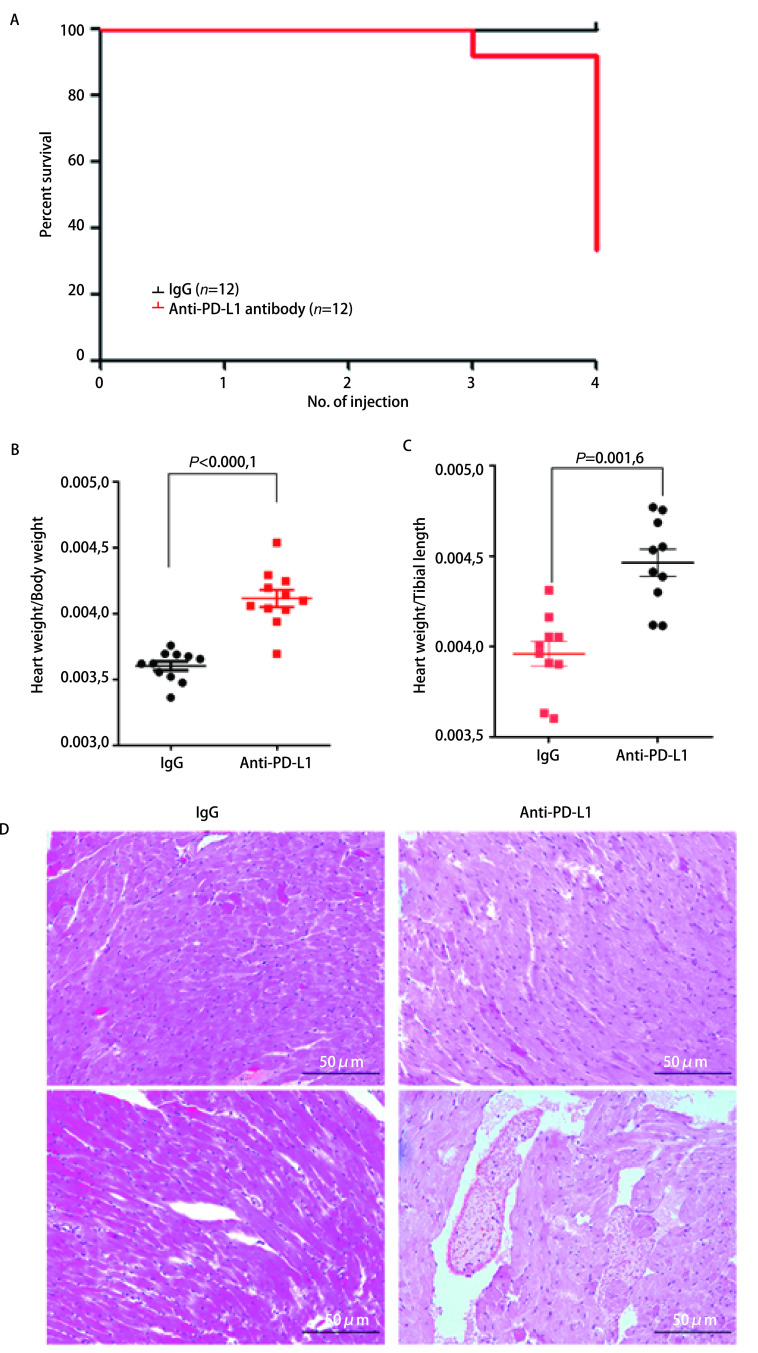
多次尾静脉注射抗PD-L1抗体导致小鼠生存期缩短和心肌非化脓性炎症。A：注射抗PD-L1抗体对小鼠生存时间的影响；B、C：抗PD-L1抗体治疗组小鼠的心重/体重比（B）和心重/胫骨长（C）比均高于对照组；D：小鼠接受抗PD-L1治疗后心肌细胞形态发生明显变化。 Multiple doses of anti-PD-L1 antibody causes mortality and non-suppurative myocarditis in recipient mice. A: The effect of anti-PD-L1 antibody on the overall survival of the mice; B, C: The heart weight/body weight ratio (B) and heart weight/tibial length ratio (C) of mice in the anti-PD-L1 antibody treated group were higher than those in the control group; D: Mice treated with anti-PD-L1 showed significant changes in cardiomyocyte morphology.

### 2.4 抗PD-L1抗体能引起小鼠心脏射血功能下降

为了进一步验证抗PD-L1抗体对小鼠心脏功能的影响，我们在连续给予4次-5次抗小鼠PD-L1抗体至小鼠后，对小鼠的血压与心肌收缩功能进行了检查。通过超声心动仪对注射抗体后小鼠的心脏射血功能进行检测（图 3A），在注射抗体15 min后，小鼠心脏射血分数（ejection fractions, EF）、左心室短轴缩短率（fractional shortening, FS）和左心室舒张末期容积（left ventricular end-diastolic volume, LVEDV）均显著降低（图 3B），表明小鼠心脏收缩减弱，射血功能下降。同时，小鼠心率加快（图C）。此外，通过鼠尾动脉测压仪测量小鼠血压发现，相较于IgG组，小鼠注射抗PD-L1抗体后收缩压和舒张压均显著降低（图 3D），且随着注射次数增加，变化趋势愈发明显，说明抗PD-L1抗体对小鼠心脏的毒性可能有一定的累积性。以上结果说明，多次静脉注射抗PD-L1抗体能明显引起小鼠心脏射血功能的下降。

**图 3:**
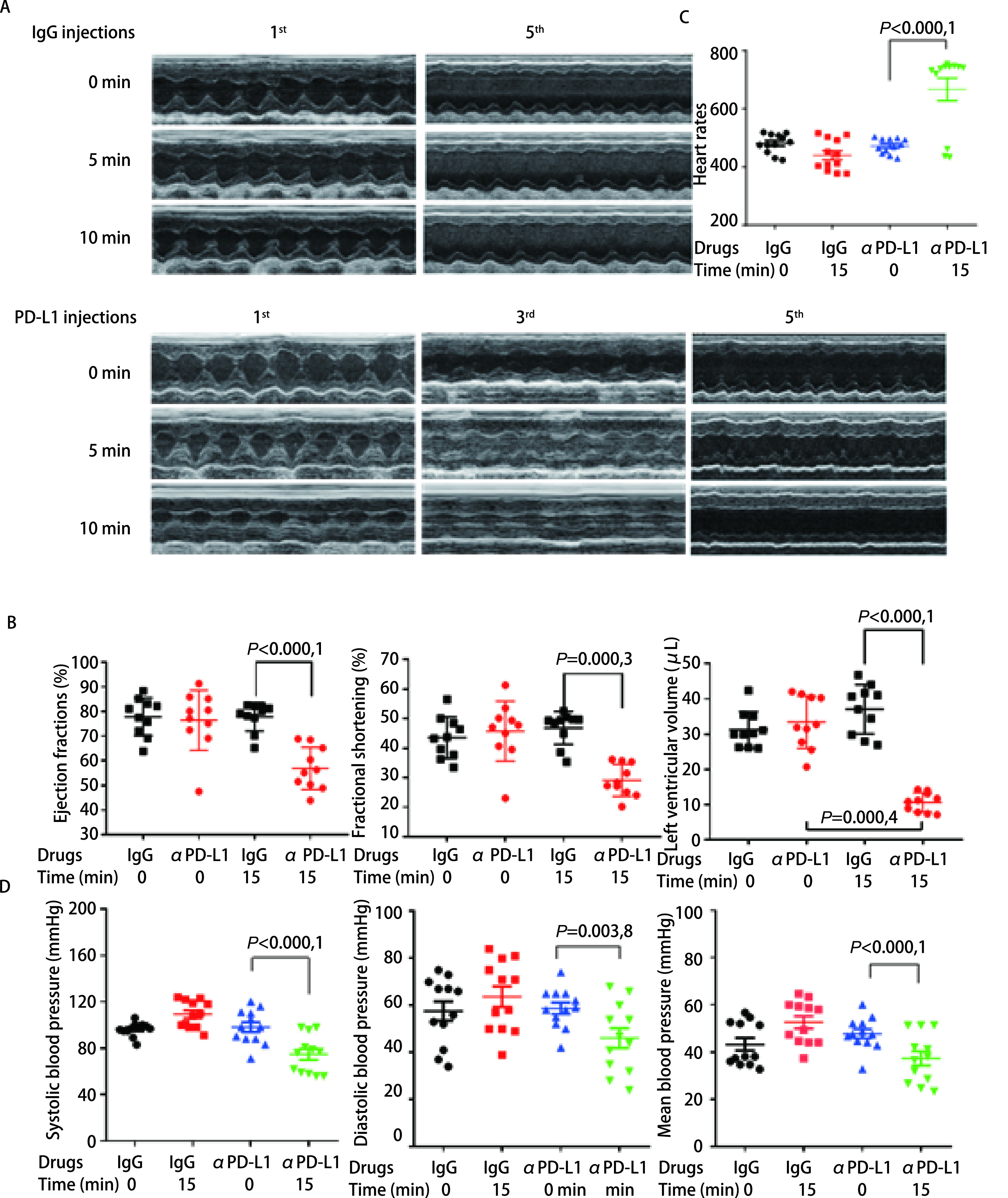
多次尾静脉注射抗PD-L1抗体导致小鼠心脏射血功能下降。A：小鼠在注射抗PD-L1单抗0 min、5 min、10 min后的超声心动图；B：注射抗PD-L1单抗的小鼠射血分数、分数缩短率和左室舒张末期容积显著降低；C：心率变化；D：小鼠血压变化 Multiple injections of anti-PD-L1 antibody results in decreased cardiac ejection in mice. A: Echocardiograms of the mice 0, 5 and 10 min after injection of anti-PD-L1 antibody; B: Ejection fraction, fractional shortening and left ventricular end-diastolic volume of the mice; C: Heart rate of the mice; D: Systolic, diastolic, and mean blood pressure of the mice.

### 2.5 抗PD-L1抗体引起小鼠心电传导异常

在验证抗PD-L1抗体对小鼠心脏射血功能的影响的同时，我们也监测了多次给予抗小鼠PD-L1抗体后，其对小鼠心电传导的影响。结果表明，在给药前与给药15 min后，注射抗PD-L1抗体的小鼠心电图发生显著变化（图 4），小鼠ECG显示ST段上斜型压低，TP段出现异常波动，提示心脏前壁和外膜下可能已经出现缺血，从而导致小鼠心肌收缩力受损。

**图 4:**
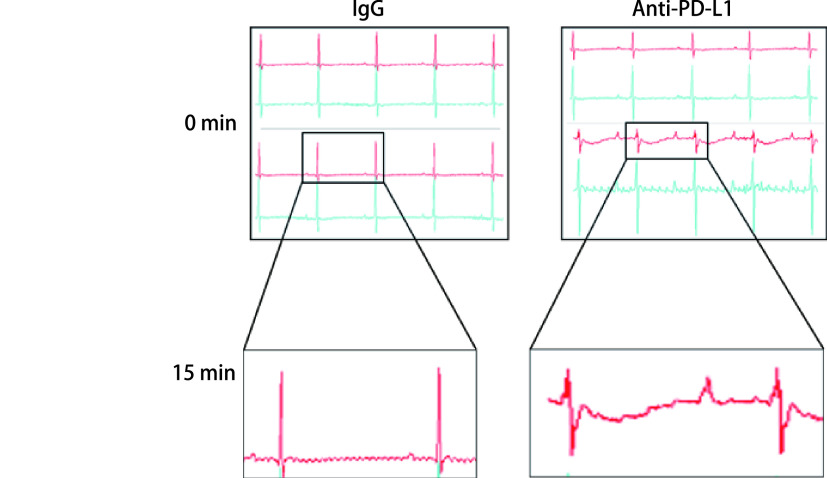
小鼠注射抗体后的心电传导异常 Electrocardiographic images of mice treated with anti-PD-L1 antibody

### 2.6 抗PD-L1抗体引起小鼠体温调节功能异常

小鼠在注射抗小鼠PD-L1抗体后，除了其心脏组织表现出明显的异常外，还出现颤抖、萎靡等表现，其体温也表现出了明显的变化。当给予抗小鼠PD-L1抗体后，小鼠体温显著降低，30 min逐渐恢复正常体温，但其静息体温较IgG组则无显著变化（图 5A）。小鼠的体温主要由垂体-下丘脑-甲状腺轴调节。免疫检查点抑制剂治疗能导致甲状腺功能受损。因此，我们检测了小鼠甲状腺功能的相关指标，发现抗PD-L1抗体组小鼠血清中的游离T4含量较IgG组显著降低（图 5B），说明抗PD-L1抗体能抑制小鼠甲状腺素的分泌，从而负反馈调节小鼠的体温。

**图 5:**
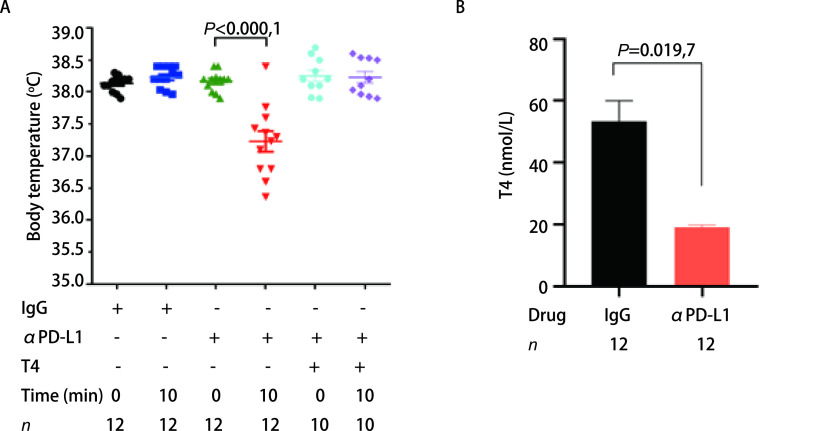
多次尾静脉注射抗PD-L1抗体引起小鼠体温降低及游离T4水平下降。A：注射抗PD-L1抗体的小鼠体温显著降低，补充甲状腺素后体温恢复正常；B：注射抗PD-L1抗体的小鼠游离T4水平较对照组低。 Multiple injections of anti-PD-L1 antibody causes a decrease in body temperature and free T4 levels in mice. A: Body temperature of mice injected with anti-PD-L1 antibody; B: Serum free T4 levels of mice injected with anti-PD-L1 antiboy. T4: tetraiodothyronine.

### 2.7 L-甲状腺素显著抑制抗PD-L1抗体引起的小鼠死亡

由于抗PD-L1抗体注射后，小鼠出现的明显症状是寒颤和体温降低，且血清中游离T4明显降低，我们因此提出，补充甲状腺素能否拯救小鼠免于死亡。我们于是在注射抗PD-L1抗体前30 min预先通过腹腔注射0.25 μg/g L-甲状腺素，发现与单用抗PD-L1抗体组小鼠相比，L-甲状腺素的使用可显著抑制小鼠体温的降低（图 5B），小鼠的不活跃状态得到了很大的缓解，且无小鼠死亡（图 6）。这些结果说明，L-甲状腺素可拮抗抗PD-L1抗体的毒性，使小鼠死亡率降低。

**图 6:**
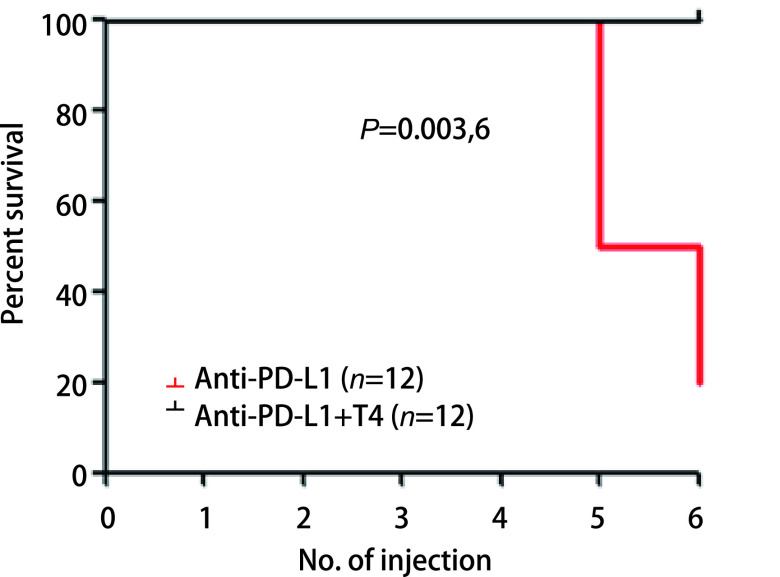
补充L-甲状腺素可显著降低抗PD-L1抗体引起的小鼠死亡率 Levothyroxine significantly reduces anti-PD-L1 antibody-induced mortality in mice

## 3 讨论

目前，抗PD-L1的抗体Atezolizumab、Durvalumab（MEDI4736）与Avelumab均已被FDA批准用于多种癌症的临床治疗，且取得了较好的治疗效果^[15-17]^。与具有严重全身副作用的免疫检查点CTLA-4抑制剂相比，阻断PD-1/PD-L1相互作用所出现的临床不良反应相对较少^[5]^。然而在临床应用中，使用抗PD-1/PD-L1抗体也会出现多种不良反应，较典型的irAEs往往出现于皮肤、胃肠道、肝脏与内分泌系统^[18]^，也会影响眼和大脑组织等免疫豁免区域^[19-21]^。而在各种免疫检查点抑制剂的应用中，抑制剂对心脏的不良反应近来引起了广泛关注。在一项抗PD-L1抗体的I期临床试验中，有1例患者用药后出现了明显的心肌炎；而抗PD-1抗体Pembrolizumab与CTLA-4抗体Ipilimumab引起心脏损伤也已有报道^[4, 22, 23]^。尽管免疫检查点阻断剂所引起的心脏的不良反应的报道相对较少，但一旦诱发产生心脏损伤，其后果往往较为严重。在近年的报道中，严重的心脏不良反应发生率有所上升：在2012年-2015年仅报道了18例，2016年报道了70例，而2017年报道了162例^[9]^。在250例发生心肌炎的患者中，死亡率在不同年龄、性别、治疗方案（抗PD-1/PD-L1或抗CTLA-4）及报告年份的患者中基本相同。心脏不良反应的发生往往具有暴发性，发展较为迅速，并经常伴随着与危重病相关的并发症，而这些并发症同样可能成为最直接的死亡原因^[24]^。相对于心脏不良反应的高死亡率及严重性，对它的研究则略显不足。除了临床用药时的警告和不良反应发生后的补救治疗外，对心脏不良反应的基础研究与动物建模也应逐步开展。

甲状腺功能受损是较为常见的不良反应。在PD-1或PD-L1抑制剂的临床试验中，甲状腺功能障碍的发生率一般在4%-19.5%之间^[25]^。而在一项Pembrolizumab治疗非小细胞肺癌的临床研究中，7.8%的患者发生了甲状腺功能减退^[14]^。

本研究中，抗PD-L1抗体对小鼠的影响大致可分为两个时期：一是注射后约1 h内的急性期，在这段时间内，小鼠表现出明显的不活跃、蜷缩不动的状态，心脏功能受到较显著的影响，包括心脏射血功能下降和心电传导异常，体温显著下降，小鼠死亡一般出现在此时期。当小鼠能渡过此急性毒性期后，小鼠的状态和体温能够恢复到正常；这些变化虽然没有引起小鼠急性的毒性反应，但这些心脏异常反应和体温异常变化可能对小鼠造成慢性毒性。此外，通过L-甲状腺素的补充可以有效缓解由抗PD-L1抗体引起的急性症状，并显著降低抗PD-L1抗体引起的死亡率，说明甲状腺功能障碍可能也是抗PD-L1抗体引起小鼠死亡的重要原因之一。另外，抗PD-L1抗体可引起小鼠致命性心肌炎，而抗PD-1抗体不会引起，说明小鼠心肌细胞表达的PD-L1在其中可能发挥重要作用。详细的作用机制值得进一步深入研究。

自从将irAEs描述为一种新的临床实体以来，人们对在小鼠中建立irAEs模型的兴趣日益浓厚，以期开发出克服这些毒副作用的方法，从而促进癌症免疫治疗的发展^[26]^。然而动物模型的研究进展却较为缓慢，其原因可能是由于小鼠肿瘤模型与免疫系统与人类的差异较大；另一方面，小鼠通常对irAE具有更强的抵抗力，因此难以找到能够真实再现irAEs的方法。本项研究的局限性在于，小鼠所使用的抗PD-L1抗体与临床上所应用的PD-L1抑制剂不尽相同，抗PD-L1抗体导致的免疫相关不良反应是否具有明显的药物特异性则仍有待研究。因此本文小鼠实验中由抗PD-L1抗体所表现的对小鼠心脏的不良反应和体温过低症状对临床指导用药仅作一定的参考借鉴。

目前，免疫治疗导致的甲状腺功能障碍的具体机制尚未探明。而抗PD-L1抗体导致的甲状腺功能减退说明抑制PD-1/PD-L1的免疫调节极有可能是原发性甲减发生的原因之一。因此，本研究结果表明，在应用PD-1/PD-L1抑制剂的肿瘤免疫治疗的同时，应该警惕其所导致的心脏损伤以及其对神经免疫-神经-内分泌网络的内环境稳态的负调节作用。

## References

[1] Zou W, Wolchok JD, Chen L (2016). PD-L1 (B7-H1) and PD-1 pathway blockade for cancer therapy: Mechanisms, response biomarkers, and combinations. Sci Transl Med.

[2] Zinzani PL, Ribrag V, Moskowitz CH (2017). Safety and tolerability of pembrolizumab in patients with relapsed/refractory primary mediastinal large B-cell lymphoma. Blood.

[3] Postow MA, Sidlow R, Hellmann MD (2018). Immune-related adverse events associated with immune checkpoint blockade. N Engl J Med.

[4] Hodi FS, O'Day SJ, McDermott DF (2010). Improved survival with ipilimumab in patients with metastatic melanoma. N Engl J Med.

[5] Topalian SL, Taube JM, Anders RA (2016). Mechanism-driven biomarkers to guide immune checkpoint blockade in cancer therapy. Nat Rev Cancer.

[6] Topalian SL, Drake CG, Pardoll DM (2015). Immune checkpoint blockade: a common denominator approach to cancer therapy. Cancer Cell.

[7] Keir ME, Butte MJ, Freeman GJ (2008). PD-1 and its ligands in tolerance and immunity. Annu Rev Immunol.

[8] Stewart R, Morrow M, Hammond SA (2015). Identification and characterization of MEDI4736, an antagonistic anti-PD-L1 monoclonal antibody. Cancer Immunol Res.

[9] Moslehi JJ, Salem JE, Sosman JA (2018). Increased reporting of fatal immune checkpoint inhibitor-associated myocarditis. Lancet.

[10] Al-Kindi SG, Oliveira GH (2018). Reporting of immune checkpoint inhibitor-associated myocarditis. Lancet.

[11] Hino R, Kabashima K, Kato Y (2010). Tumor cell expression of programmed cell death-1 ligand 1 is a prognostic factor for malignant melanoma. Cancer.

[12] Hamanishi J, Mandai M, Iwasaki M (2007). Programmed cell death 1 ligand 1 and tumor-infiltrating CD8^+^ T lymphocytes are prognostic factors of human ovarian cancer. Proc Natl Acad Sci U S A.

[13] Keir ME, Liang SC, Guleria I (2006). Tissue expression of PD-L1 mediates peripheral T cell tolerance. J Exp Med.

[14] Reck M, Rodriguez-Abreu D, Robinson AG (2016). Pembrolizumab versus chemotherapy for PD-L1-positive non-small-cell lung cancer. N Engl J Med.

[15] Rosenberg JE, Hoffman-Censits J, Powles T (2016). Atezolizumab in patients with locally advanced and metastatic urothelial carcinoma who have progressed following treatment with platinum-based chemotherapy: a single-arm, multicentre, phase 2 trial. Lancet.

[16] Syed YY (2017). Durvalumab: First global approval. Drugs.

[17] Kaufman HL, Russell J, Hamid O (2016). Avelumab in patients with chemotherapy-refractory metastatic Merkel cell carcinoma: a multicentre, single-group, open-label, phase 2 trial. Lancet Oncol.

[18] Postow MA (2015). Managing immune checkpoint-blocking antibody side effects. Am Soc Clin Oncol Educ Book.

[19] Crews J, Agarwal A, Jack L (2015). Ipilimumab-associated retinopathy. Ophthalmic Surg Lasers Imaging Retina.

[20] Henderson AD, Thomas DA (2015). A case report of orbital inflammatory syndrome secondary to ipilimumab. Ophthalmic Plast Reconstr Surg.

[21] Bossart S, Thurneysen S, Rushing E (2017). Case report: Encephalitis, with brainstem involvement, following checkpoint inhibitor therapy in metastatic melanoma. Oncologist.

[22] Brahmer JR, Tykodi SS, Chow LQ (2012). Safety and activity of anti-PD-L1 antibody in patients with advanced cancer. N Engl J Med.

[23] Laubli H, Balmelli C, Bossard M (2015). Acute heart failure due to autoimmune myocarditis under pembrolizumab treatment for metastatic melanoma. J Immunother Cancer.

[24] Mahmood SS, Fradley MG, Cohen JV (2018). Myocarditis in patients treated with immune checkpoint inhibitors. J Am Coll Cardiol.

[25] Cukier P, Santini FC, Scaranti M (2017). Endocrine side effects of cancer immunotherapy. Endocr Relat Cancer.

[26] Fecher LA, Agarwala SS, Hodi FS (2013). Ipilimumab and its toxicities: a multidisciplinary approach. Oncologist.

